# A commentary on ‘Inflammatory biomarkers and nanotechnology: new insights in pancreatic cancer early detection’

**DOI:** 10.1097/JS9.0000000000000633

**Published:** 2023-07-31

**Authors:** Ruiyuan Xu, Chengcheng Wang, Yupei Zhao

**Affiliations:** a Department of General Surgery; b State Key Laboratory of Complex Severe and Rare Diseases; c Medical Science Research Center, Peking Union Medical College Hospital, Chinese Academy of Medical Sciences, Peking Union Medical College; d Key Laboratory of Research in Pancreatic Tumor, Chinese Academy of Medical Sciences; e National Science and Technology Key Infrastructure on Translational Medicine in Peking Union Medical College Hospital, Beijing, People’s Republic of China

*Dear Editor*,

We read with great interest the study entitled “Inflammatory biomarkers and nanotechnology: new insights in pancreatic cancer early detection”^1^, which indicated that a multiplexed analysis combining a magnetic levitation (MagLev)-based approach and common inflammatory biomarkers could discriminate pancreatic ductal adenocarcinoma (PDAC) patients from healthy controls. The findings provide critical value for the early detection of pancreatic cancer. However, some concerns need to be discussed.

First, there are some inconsistencies in patient data. For instance, the results showed that the median age of non-oncological patients (NOPs) and PDAC patients was 64.5 years (interquartile range: 46–74) and 73 years (interquartile range: 64–79), respectively. However, the median age of healthy controls and PDAC patients was 58.5 years (range from 23 to 84) and 71 years (range from 47 to 83), respectively, in supplementary Table S1 (see^1^). Similarly, in the results section, 13 (60%) female and 9 (40%) male subjects were revealed in NOPs, and 13 (54%) female and 11 (46%) male patients were shown in the PDAC group. Nevertheless, the supplementary Table S1^1^ showed that there were 10 (46%) female and 12 (54%) male subjects in NOPs, and 9 (37.5%) female and 15 (62.5%) male patients were shown in the PDAC group. Considering the contradictions in characteristics among patients, the authors are advised to carefully check and verify the data to provide accurate patient characteristics.

Second, the baseline clinicopathological features of PDAC patients and healthy controls were not comprehensive. Some potential confounding factors affecting the inflammatory state between NOPs and PDAC patients were not included, such as body mass index, history of alcohol intake, and the levels of alanine transaminase (ALT) as well as aspartate transaminase (AST). Notably, in terms of smoking status, the ex-smokers and current smokers in the PDAC group account for 80%, while the number in healthy controls was only 50%, which may cause unbalanced baseline characteristics. Previous studies have indicated that cigarette smoking contributes to the state of pulmonary inflammation and the associations between neutrophil-to-lymphocyte ratio (NLR) as well as the platelet-to-lymphocyte ratio (PLR) and chronic obstructive pulmonary disease (COPD) have been demonstrated^2,3^. Hence, the upregulation of NLR, derived-NLR (d-NLR), and PLR in the PDAC group could be partially correlated with smoking status, resulting in an underlying bias of results.

Third, although immune-suppressive inflammation is strongly associated with the progression of PDAC, it should be noted that the inflammation state and phenotypes of immune cells in peripheral blood were not very consistent with those in peritumoral or intratumoral tissue. An elegant study performed by Daley *et al*.^4^ showed that a dominant PDAC-infiltrating lymphocyte population, γδT cells, represented with distinct numbers and subsets compared with their counterparts in peripheral blood mononuclear cells (PBMC). Thus, the multiplexed approach combining nanosystems with inflammatory biomarkers to investigate the inflammation state and further identify high-risk PDAC patients in peri-pancreatic or intra-pancreatic tissue is recommended for evaluation. Furthermore, multiple serum markers such as CA19‐9, specific autoantibodies, and glycoprotein biomarkers for the early diagnosis of PDAC have been identified^5^. The synergistic analysis that combines nanotechnology, inflammatory markers, and other identified serum markers needs to be verified in further studies.

In conclusion, the study provides valuable evidence for the early detection of pancreatic cancer by combining inflammatory biomarkers and a user-friendly nanotechnology approach. However, the multiplexed strategy needs to be further validated in larger clinical studies. Moreover, the pathogenesis of PDAC develops from well-defined precursor lesions such as pancreatic intraepithelial neoplasia (PanIN) and intraductal papillary mucinous neoplasm (IPMN). Further studies focusing on patients with pancreatic precancerous lesions using the multiplexed approach may bring greater clinical benefits.

## Ethical approval

Not applicable.

## Consent

Not applicable.

## Sources of funding

This work was supported by the CAMS Innovation Fund for Medical Sciences (2021-I2M-1-002) and the National Natural Science Foundation of China (82102810).

## Author contribution

R.X., C.W., and Y.Z.: conceptualization; R.X.: writing – original draft; R.X. and C.W.: writing – review and editing; C.W. and Y.Z.: funding acquisition.

## Conflicts of interest disclosure

The authors declare that they have no conflicts of interest.

## Research registration unique identifying number (UIN)

Not applicable.

## Guarantor

Yupei Zhao.

## Data availability statement

Not applicable.

## Provenance and peer review

Our paper was not invited.
